# Are general wards sufficiently staffed to care for level 1 patients?

**DOI:** 10.1186/cc12444

**Published:** 2013-03-19

**Authors:** G Rajendran, C Tjen, S Hutchinson, S Fletcher

**Affiliations:** 1 Norfolk & Norwich University Hospital, Norwich, UK

## Introduction

There are several definitions of level 1 (L1) care, all refer to a group at risk of clinical deterioration on the ward [1-3]. There is evidence that ward patients who become acutely unwell often receive suboptimal care [4]. A regional study commissioned by Norfolk, Suffolk & Cambridgeshire Critical Care Network (NSCCCN) found that a majority of ward patients may be of L1 dependency and death rates appear to be correlated with L1 status. We aim to examine the relationship between the ward distribution of illness acuity, staffing and patient outcome.

## Methods

Data were collected as part of NSCCCN's observational prevalence study in 2010. Ward surveys included acuity of illness, staffing levels and skill mix. Secondary data were obtained from the Patient Administration System. Emergency, oncology, paediatric and maternity units were excluded.

## Results

Complete datasets were obtained from 1,402 patients in 22 wards in our university hospital over two seasons. This constitutes 98.3% of inpatients from those wards. The mean ward occupancy rate was 94% (10th to 90th percentile: 85% to 100%). At least one L1 acuity criterion was scored by 898 (64%) patients, with 25% from geriatrics followed by orthopaedics (17%) and general surgery (10%). Each ward had an average of eight qualified nursing staff (range: 4 to 12) equating to an average staff:patient ratio (SPr) of 0.253. There was no correlation between ward occupancy and nursing staff (Pearson correlation, corr: 0.55), nor between prevalence of L1 criteria and staffing (corr: 0.34). The admission rate to intensive care was noted to be higher if the patients were nursed in a ward with lower than average SPr compared with higher SPr (2.7% vs. 1.2%, *P *= 0.058 Fisher's exact), but this was not statistically significant. Senior nursing (Band 6) staff were part of the skill mix on only nine of 44 ward surveys.

## Conclusion

Better outcome with improved SPr may be unsurprising, although if proven conclusively would significantly inform workforce planning. Lack of correlation between staffing levels and occupancy or acuity is also interesting given that we know L1 criteria are associated with worse outcome.

## References

[B1] Levels of Critical Care for Adult Patients 2002 Intensive Care Society http://www.ics.ac.uk/professional/standards_safety_quality/standards_and_guidelines/levels_of_critical_care_for_adult_patients

[B2] Acutely Ill Patients in Hospital 2007 NICE Guideline 50. NICE http://www.nice.org.uk/cg50

[B3] AUKUH Acuity/Dependency Tool 2007 Association of UK University Hospitals http://www.aukuh.org.uk/index.php/affiliate-groups/directors-of-nursing/patient-care-portfolio

[B4] An Acute Problem? 2005 NCEPOD http://www.ncepod.org.uk/2005report/index.html

